# Circulating MicroRNA Panel as a Diagnostic Marker for Hepatocellular Carcinoma

**DOI:** 10.5152/tjg.2022.21183

**Published:** 2022-10-01

**Authors:** Xiaochang Wu, Renrui Wan, LingYan Ren, Yong Yang, Yuan Ding, Weilin Wang

**Affiliations:** 1 Department of Hepatobiliary and Pancreatic Surgery, The Second Affiliated Hospital, Zhejiang University School of Medicine, Hangzhou, China; 2 Department of Hepatobiliary Surgery, Huzhou Central Hospital, Zhejiang University Huzhou Hospital, Huzhou, Zhejiang, China; 3 Department of Nephrology, the First People’s Hospital of Huzhou, the First Affiliated Hospital of Huzhou Teachers College, Huzhou, Zhejiang, China; 4 Clinical Medicine Innovation Center of Precision Diagnosis and Treatment for Hepatobiliary and Pancreatic Disease of Zhejiang University, Hangzhou, China; 5 Key Laboratory of Precision Diagnosis and Treatment for Hepatobiliary and Pancreatic Tumor of Zhejiang Province, Hangzhou, China; 6 Research Center of Diagnosis and Treatment Technology for Hepatocellular Carcinoma of Zhejiang Province, Hangzhou, China; 7 Clinical Research Center of Hepatobiliary and Pancreatic Diseases of Zhejiang Province, Hangzhou, China

**Keywords:** Hepatocellular carcinoma, microRNA, plasm biomarkers

## Abstract

**Background:**

Current diagnostic markers for hepatocellular carcinoma are compromised and limited by their low sensitivity and specificity. In this study, circulating microRNAs were utilized as a diagnostic tool to segregate hepatocellular carcinoma patients from healthy subjects.

**Methods:**

We analyzed 2 public datasets for differences in plasma microRNA expression profiles of hepatocellular carcinoma patients and healthy controls to identify biomarkers related to hepatocellular carcinoma. Plasma samples from hepatocellular carcinoma patients and control subjects were then collected for next-generation microRNA sequencing analysis. The differential microRNAs obtained from the above 3 parts were intersected to obtain microRNAs that were significantly different between the 2 groups. We then analyzed 58 specimens, which come from hepatocellular carcinoma and the control group, for validation through a quantitative polymerase chain reaction. The diagnostic value of these differentially expressed miRNAs was assessed by receiver operating characteristic curve analysis.

**Results:**

The levels of miR-206 and miR-222 were significantly higher (*P *< .05) and the level of miR-126 was lower *(P *< .05) in patients with hepatocellular carcinoma than in healthy subjects. Receiver operating characteristic analysis established a powerful diagnostic accuracy when miR-206, miR-222, and miR-126 were combined (area under curve = 0.887), which was similar to that of the marker α-fetoprotein (area under curve = 0.889). When the microRNAs were combined with α-fetoprotein, the accuracy of hepatocellular carcinoma diagnostic potential was further improved (area under curve = 0.989).

**Conclusion:**

We identified 3 microRNAs significantly altered in the plasma of hepatocellular carcinoma patients and they can screen patients at risk of hepatocellular carcinoma.

## Main Points

- We identified 3 microRNAs (miRNAs) (miR-206, miR-126, and miR-222) that could be used to screen patients at risk of hepatocellular carcinoma (HCC).
- The 3 miRNAs have a powerful diagnostic accuracy when they were combined.
- By combining the panel of 3 miRNAs and α-fetoprotein, the specificity and sensitivity of HCC detection will be further improved.

## Introduction

The rate of hepatocellular carcinoma (HCC) incidence ranks fourth among all tumors and its mortality rate ranks third.^1^ A total of 905 677 new cases of liver cancer and 830 180 deaths were reported globally in 2020, of which more than half of the deaths came from China.^2^ Since patients with early HCC have no obvious clinical symptoms, early diagnosis of liver cancer is very difficult and insufficient. At present, screening for liver cancer mainly relies on imaging, such as computed tomography, magnetic resonance imaging, and tumor markers, especially α-fetoprotein (AFP). However, these screening methods still have major shortcomings, such as a low AFP-positive rate for early liver cancer. For the early stage of HCC, approximately 40% of AFP was negative. For advanced HCC, the rate was 25%.^3^ The practice guidelines of the American Association for the Study of Liver Diseases have excluded AFP as a diagnostic marker for HCC, and support for this change has been confirmed in other related studies.^4,5^ In addition, pathological tests are invasive and can lead to complications such as bile leaks, bleeding, and infection. These shortcomings suggest that if we want to improve the early diagnostic rate of HCC patients, we must find new and more reliable diagnostic markers.

MicroRNAs (miRNAs) play an important role in the development and progression of HCC^6-8^ by regulating the expression of target genes.^9,10^ Increasingly, miRNA profile analysis has shown significantly dysregulated miRNAs in human malignant tumors. Many miRNAs are related to tumor occurrence, progression, and even tumor response to treatment. Even under unfavorable conditions, such as low temperature or low pH, the physical properties and expression of miRNAs are very stable in various body fluids, including plasma, serum, and urine.^11^ Moreover, miRNA expression levels are easy to estimate and some even demonstrate tissue specificity.^12^ These characteristics of miRNAs make them prime candidates for early cancer diagnosis and treatment monitoring.

Based on the above characteristics of miRNAs, we hypothesized that developing a diagnostic platform based on the combination of multiple miRNAs would improve the sensitivity and specificity of HCC detection. Herein, we carried out a systematic and comprehensive miRNA biomarker detection process to establish a new HCC-specific miRNA expression profile for the early detection of HCC. Our findings were initially confirmed in an analysis of multiple large public data sets and then rigorously validated and evaluated in an independent clinical cohort.

## Materials and Methods

### Patients and Samples

Plasma samples were collected from 38 newly diagnosed primary HCC patients, from March 2019 to April 2020. At the same time, plasma from 20 healthy subjects, who were confirmed to have no HCC, was collected. All HCC patients were initially diagnosed with HCC and did not receive surgery or other treatment. Do not combine with the other tumor. In addition to chronic hepatitis B, the HCC patients do not contain other chronic diseases, such as chronic hepatitis C, autoimmune hepatitis, and so on. All the volunteers may have had chronic hepatitis infection, but no other diagnosed diseases were found. All participants signed a written consent form approved by the Ethics Committee (Approved 20200804) prior to participation. Clinical examinations were conducted, including the determination of AFP levels using the Diagnostic Kit for Alpha-fetoprotein (ELISA) according to the manufacturer’s instructions (Table 1).

### Identification of Differential HCC Plasma miRNAs in 2 Datasets

Two large public datasets (The Cancer Genome Atlas (TCGA) and GSE50013) were used to search for differential plasma miRNA expression profiles in HCC patients and healthy controls to avoid the potential bias of single-center data. The Cancer Genome Atlas miRNA expression profiling data were downloaded from the Genomic Data Commons (GDC) (https://portal.gdc.cancer.gov, accessed on April 23, 2020), (Supplementary Table 1). Likewise, GSE50013-processed miRNA expression profiling data (both normalized miRNA profiling and clinical data) were downloaded from the Gene Expression Omnibus (GEO) database (https://www.ncbi.nlm.nih.gov, accessed on May 28, 2020) (Supplementary Table 2).

### RNA Isolation and Library Construction

RNA samples were subjected to a series of strict quality-control tests. Library construction of qualified samples was performed using a NEBNext Ultra RNA Library Prep Kit for Illumina (BioLabs, New England). Qualified libraries were used for next-generation high-throughput sequencing and quantitative polymerase chain reaction (qPCR).

### Next-Generation Sequence Analysis of Plasma miRNAs

Total RNA extracted from plasma and passing quality control checks was used for high-throughput small RNA sequence analysis using an Illumina HiSeq2500 (Biomarker Technologies Corporation, Beijing, China). For miRNA identification, we compared the read sequence of the reference genome with the mature miRNA sequence in the known miRNA database miRBase (v21). Reads with the same sequence as the known miRNA were considered to be known miRNAs identified in this project. When detecting differentially expressed miRNAs, we used edgeR software for differential screening using fold change > 1.5 and* P *< .05 as the screening criteria (Supplementary Table 3).

### RNA Isolation and qPCR Validation of Plasma miRNA in Clinical Samples

Total RNA was extracted from plasma using TRIzol according to the manufacturer’s instructions (ThermoFisher Scientific, USA). Before isolation, approximately 5.6 × 10^8^ copies of *Caenorhabditis elegans* mir-39 spike-in control RNA (Qiagen, Germany) were added to each plasma sample. RNA quality was analyzed spectrophotometrically and electrophoretically. Total RNA concentration was estimated using a Nanodrop spectrophotometer (ThermoFisher Scientific) and 500 ng/2.5 μg total RNA was used for cDNA synthesis for miRNA from plasma using miScript PCR Starter Kit (Qiagen) and Revertaid reverse transcriptase (ThermoFisher Scientific), respectively.

Complementary DNA was quantified using the SYBR GREEN PCR Master Mix (ThermoFisher Scientific) and the QuantStudio 6 Flex RealTime PCR system (ThermoFisher Scientific) with gene-specific primers (Supplementary Table 4). Internal and external miRNA controls included hsa-miR-16-5p and *C. elegans* mir-39, respectively. Expression analysis was quantified using the 2^−ΔΔCt^ method.

### Statistical Analysis

For the clinical samples, receiver operating characteristic (ROC) curves and area under curve (AUC) values were determined using MedCalc (V19.0.7, MedCalc Software Ltd. Acacialaan, Ostend, Belgium) and GraphPad Prism (V8.0.2. GraphPad Software, San Diego, USA). Statistical Package for the Social Sciences 23.0 (IBM Corp.; Armonk, NY, USA) was used to analyze differences in various indicators between the 2 groups; *P *< .05 was considered statistically significant.

## Results

### Analysis of Differential miRNAs in the Plasma of HCC and Healthy Controls in Clinical Samples and Public Domain Datasets

**Figure 1.**
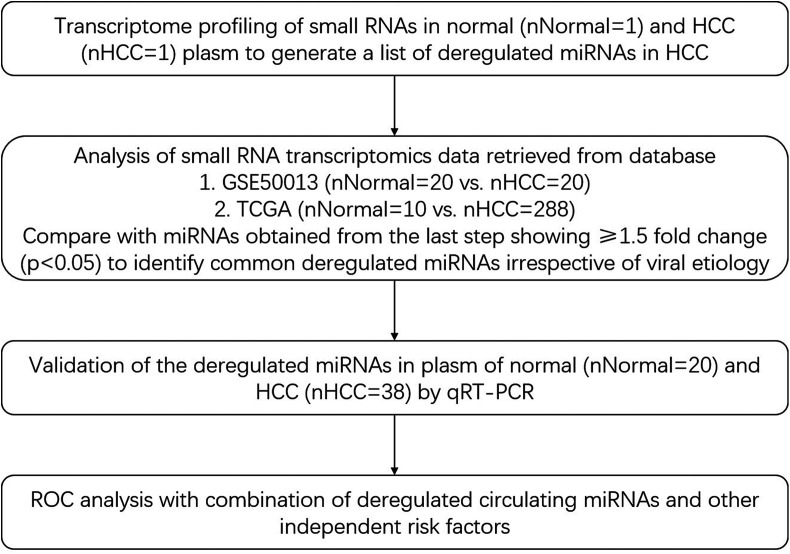
The schematic presentation of the study protocol.

**Figure 2.**
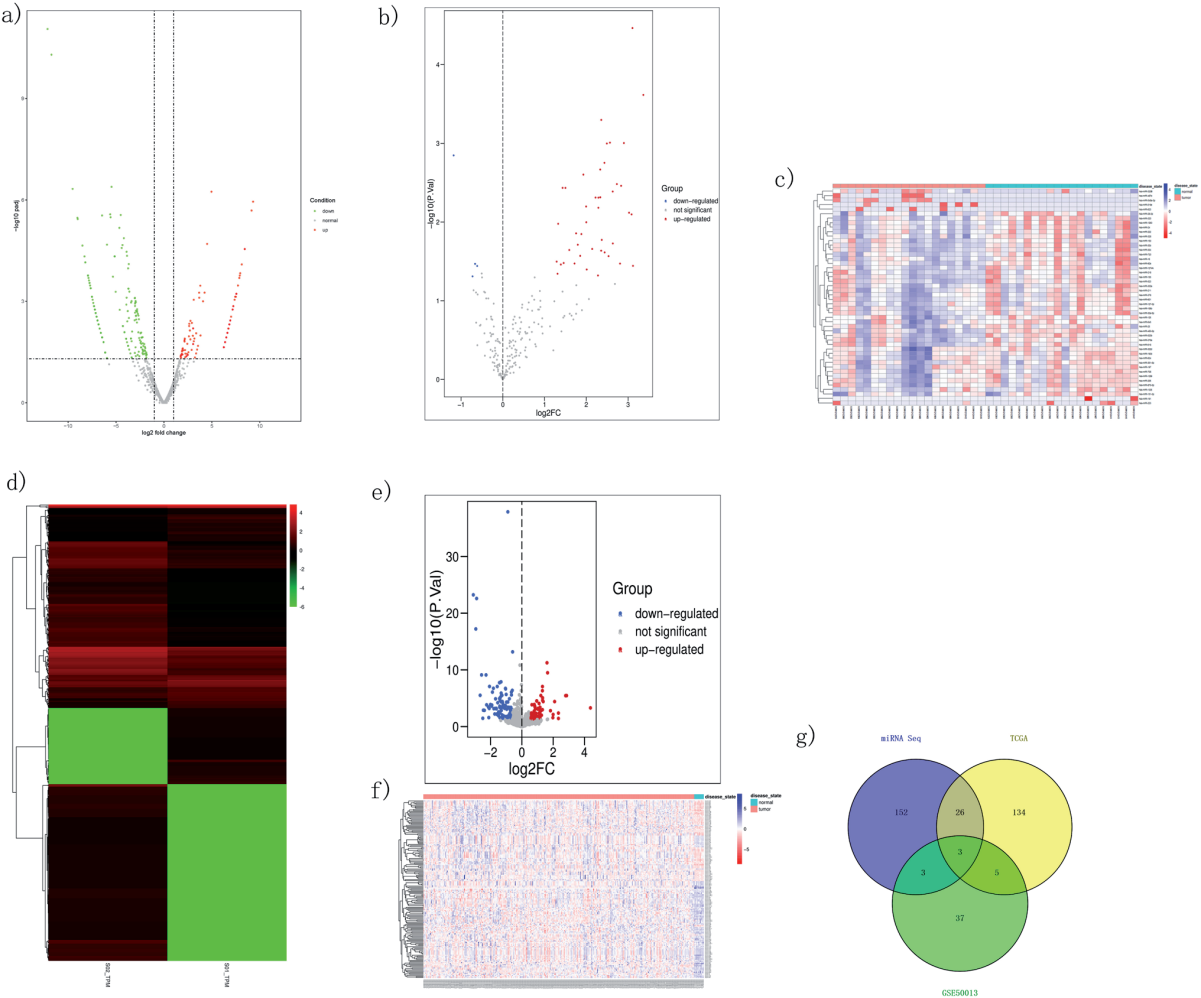
Differential gene screening. Volcano plots (A, B, and E) and hierarchical cluster maps (C, D, and F) show data of miRNAs differentially expressed in HCC and control samples. Venn diagram (G) shows that comparing the data of multiple samples, 3 HCC-related differentially expressed miRNAs (log_2_FoldChange > 1.5, *P *< .05) are found. HCC, hepatocellular carcinoma; miRNA, microRNA.

The detailed study protocol is presented in Figure 1. We used the Illumina platform to analyze the differential expression of miRNAs in the plasma of HCC (n = 1) and normal subjects (n = 1). Since these deregulated miRNAs were obtained only in a single HCC plasma sample, the differential expression data were further verified and compared with data sets disclosed in GEO and GDC. One GEO dataset, GES50013 (n = 20 for both HCC and normal subjects), revealed 48 abnormally expressed miRNAs. A similar analysis of the GDC data set TCGA revealed 170 abnormally expressed miRNAs in the plasma of HCC (n = 288) and normal (n = 10) samples. Volcano map and hierarchical cluster analysis showed the differences and degree of change in the expression of miRNAs between the HCC and normal samples (Figure 2a-f). Comparing the research results, we found 3 miRNAs (miR-206, miR-126, and miR-222) (Figure 2g) related to liver cancer.

### Validation of Deregulated miRNAs in the Plasma of HCC Patients

**Figure 3.**
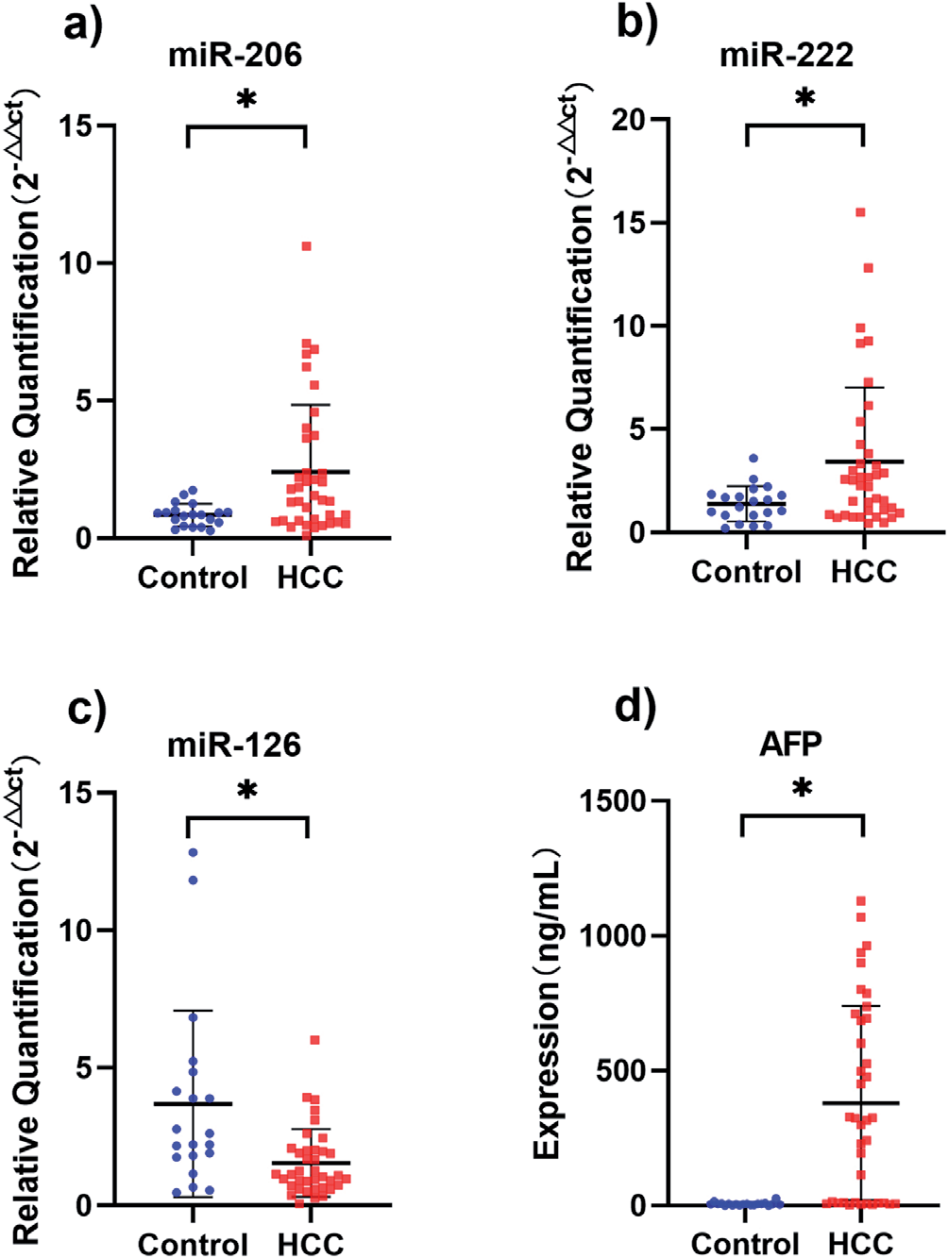
qRT-PCR analysis of miRNAs. The 2 upregulated miRNAs (A,B) and 1 downregulated miRNA (C). (D) Expression levels of AFP in HCC and the control groups. *Depict *P *< .05. qRT-PCR, quantitative reverse transcription-polymerase chain reaction; miRNA, microRNA; HCC, hepatocellular carcinoma; AFP, α-fetoprotein.

To confirm the correlation between the 3 miRNAs and HCC, we conducted a qPCR validation analysis in 58 verified patient samples (20 normal and 38 HCC patients). The results were consistent with the difference analysis results (Figure 3a-c).

### Evaluation of the Diagnostic Ability of the 3 miRNAs in HCC and Control Subjects

**Figure 4.**
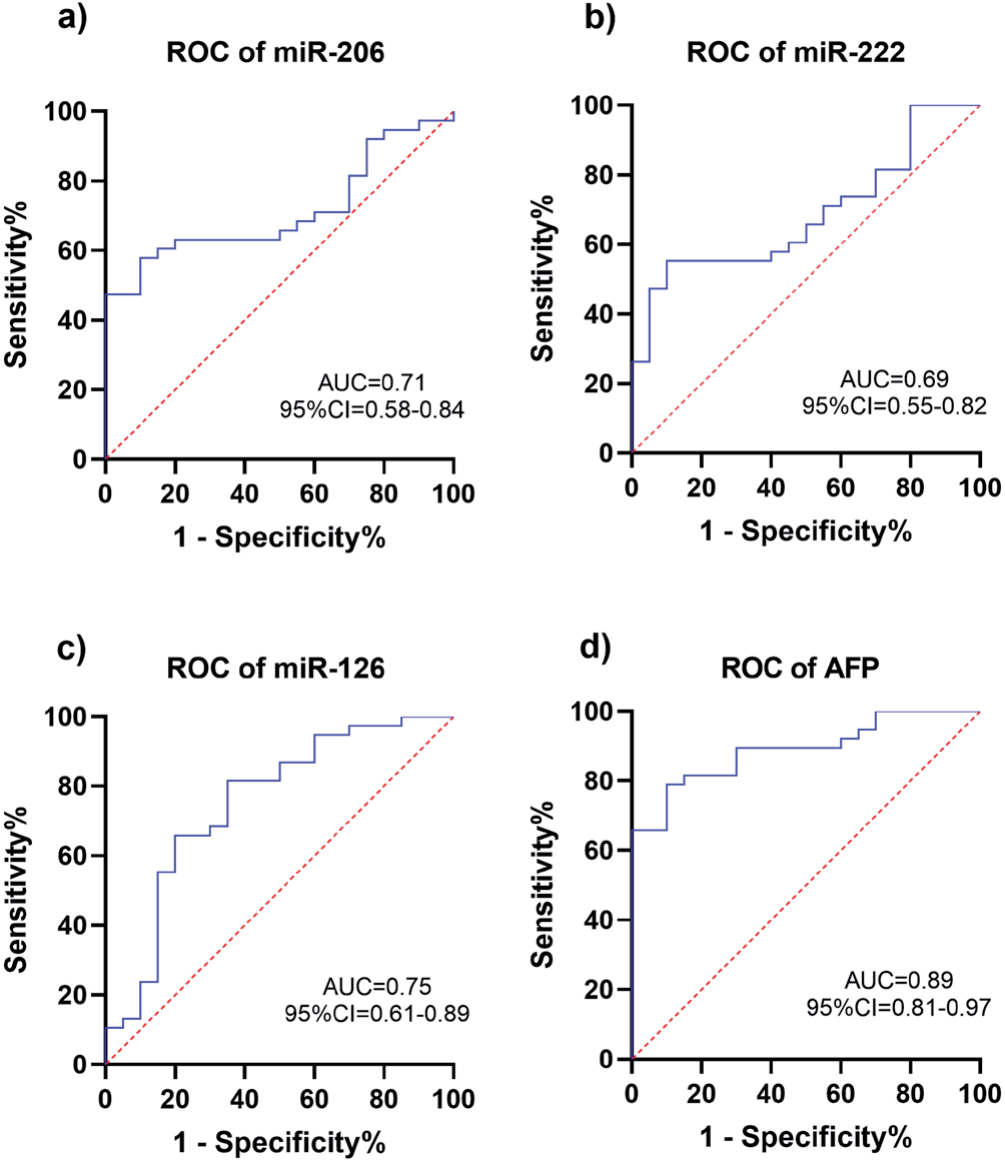
ROC analysis of the 3 miRNAs. (A) miR-206 in normal versus HCC, (B) miR-222 in normal versus HCC, (C) miR-126 in normal versus HCC, and (D) AFP in normal versus HCC. miRNA, microRNA; HCC, hepatocellular carcinoma; AFP, α-fetoprotein; ROC, receiver operating characteristic.

We used ROC curves and AUC values to evaluate the possibility of each of the 3 miRNAs serving as potential non-invasive diagnostic markers for HCC by discriminating between the experimental group and the control group (Figure 4a-c). Furthermore, the accuracy of AFP as a diagnostic marker for liver cancer was demonstrated in the clinical samples for comparison (Figure 4d).

**Figure 5.**
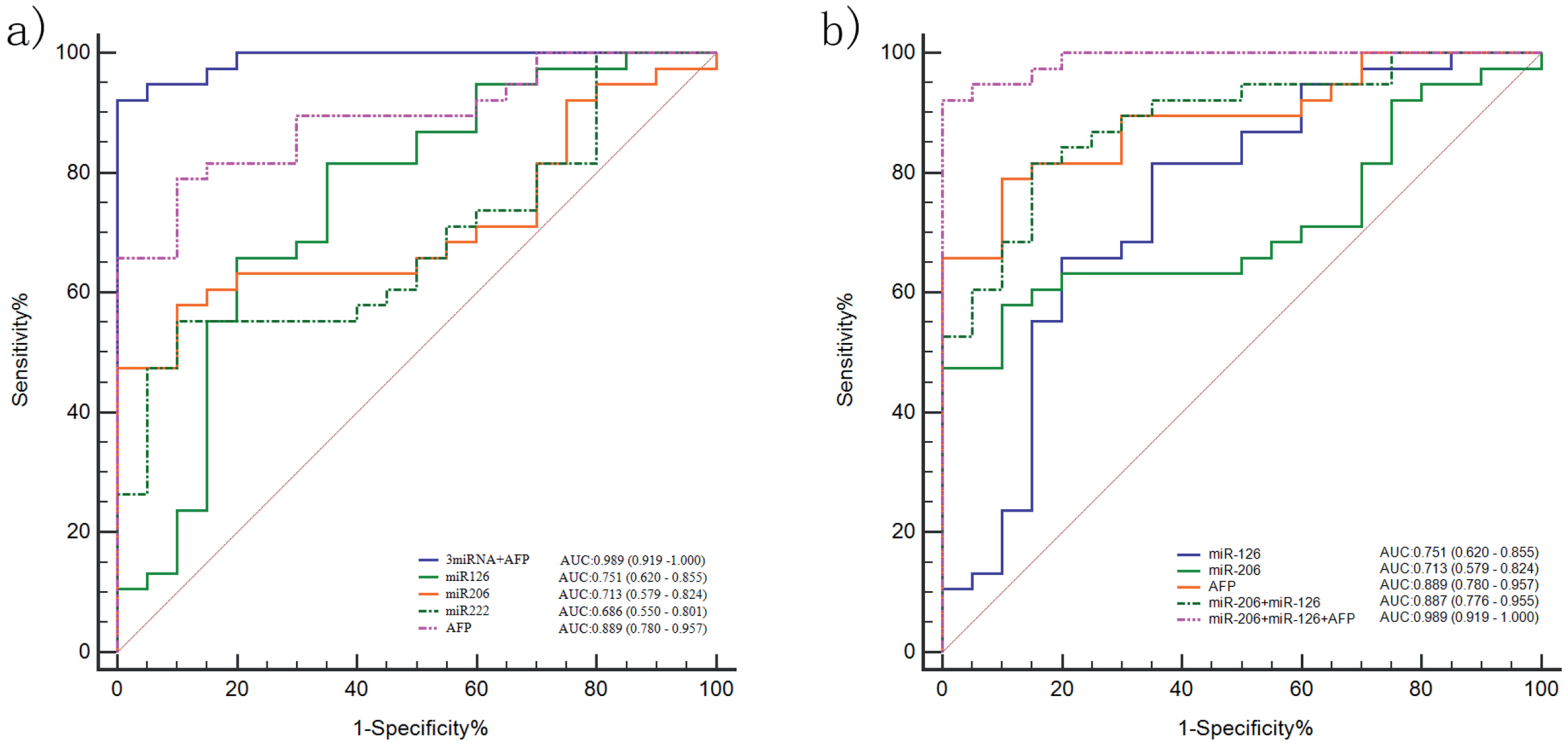
Evaluation of sensitivity and specificity between miRNA and the combination of miRNA and AFP as diagnostic markers. miRNA, microRNA; AFP, α-fetoprotein.

In an attempt to improve diagnostic accuracy, ROC curves were plotted for the combined miRNAs in HCC compared with healthy control subjects. The combination of the 3 miRNAs improved the accuracy of the miRNAs as a diagnostic marker (Figure 5). Combining AFP, a widely recognized and important HCC biomarker, with the 3 miRNAs further improved the diagnostic accuracy of HCC based on ROC curve analysis and AUC values (Table 2; Figure 5a-b).

### Evaluation of the 3 miRNAs as Independent Risk Factors for HCC

Multivariate analysis showed that high levels of miR-206 (odds ratio (OR) = 2.953, 95% CI 1.156-7.542, *P *= .024), high levels of miR-222 (OR = 1.745, 95% CI 1.036-2.940, *P *= .024), and low levels of miR-126 (OR = 0.576, 95% CI 0.381-0.8699, *P *= .009) were independent risk factors for HCC (Table 3). Combinations of miR-206 and miR-126 could also be independent risk factors for HCC development (*P *< .05). The risk score model was as follows: −0.064 + (−1.534 × miR-126) + (2.357 × miR-206) + (0.60 × miR-222).

The liver cancer diagnostic accuracy of miR-206 and miR-126 combined was similar to that of AFP based on comparative ROC curve analysis (Table 2). This suggests that these 2 miRNAs have a high diagnostic value for HCC.

## Discussion

A number of studies have shown that circulating miRNAs, such as miR-21,^13^ miR-221,^14^ and miR-125b,^15^ have a biomarker effect on the early diagnosis and prognostic detection of different cancers.^12^ The miRNAs miR-433 and miR-191 can be used as circulating biomarkers for prostate cancer and breast cancer, respectively.^16,17^ Nishiwada et al^18^ found that joint application of miRNAs improved the diagnosis of pancreatic ductal adenocarcinoma patients at risk for lymph node metastases.

In the current study, 3 HCC-related miRNAs (miR-126, miR-206, and miR-222) were assessed in the plasma of HCC patients. The median plasma levels of miR-206 and miR-222 were significantly upregulated in HCC patients, whereas miR-126 was markedly reduced. We used ROC analysis to determine the HCC diagnostic capabilities of these 3 miRNAs and observed that none of them on their own performed better than AFP in distinguishing HCC. As we all know, the use of a combination of 2 or more biomarkers overcomes the limitations of the sensitivity and specificity of a single marker to detect HCC. Therefore, we tested different combinations of miR-126, miR-222, miR-206 in the HCC diagnostic test and achieved a maximum diagnostic efficiency (miR-126 + miR-206, AUC = 0.887), which is similar to that of AFP (AUC = 0.889). When the miRNAs were combined with AFP, the accuracy of the HCC diagnostic potential was further improved (AUC = 0.989). Interestingly, during the logistic regression analysis, we found that the *P *value of miR-222 > .05 and no change in AUC value before and after the inclusion of miR-222 in the equation. This may be caused by multicollinearity.

Our study showed that compared with the control group, the level of miR-126 in the plasma of HCC patients was significantly downregulated. Zhao et al^19^ previously reported that miR-126 was downregulated in the blood of HCC patients, which confirms our findings. The Zhao et al^19^ study also showed that miR-126 induced apoptosis by targeting the Sox2 gene, exerting its anti-tumor activity. However, Faranda et al^20^ reported increased expression of miR-126 in HCC patients. The conflicting findings suggest that further studies are needed. Unlike miR-126, our results and those of a similar study^21^ showed that the plasma level of miR-222 was significantly upregulated in HCC patients. Upregulation of miR-222 has been found to induce hepatocyte proliferation by inhibiting cell cycle participant P27.^22^ However, Wang et al^23^ reported a significant reduction in miR-222 levels in patients with HCC, which is contrary to previous experimental results and this study. Our study found that the third miRNA, miR-206, was weakly expressed in the plasma of HCC patients, findings consistent with those of Mirzaei.^24^

As is evident from these various reports, the experimental results are not always consistent across studies. These inconsistencies require further refinement of the study design to produce more consistent and reliable results. Among the possible reasons for these inconsistencies are flaws in sample preparation^25^ and poorly employed methods.^26^ For example, in the process of miRNA quantification, insignificant hybridization during reverse transcription-PCR may be a source of variation.^27^ In addition, improper selection of reference genes and different experiment guidance may lead to inconsistent quantification of circulating miRNA levels in different tissues.^28^ For example, one study found that the level of miR-122 in HCC tissues and cell lines decreased, whereas, in serum, the level increased.^29^ Other studies have shown that miRNAs can be released in the form of free molecules or microvesicles, which can be detected in most biological fluids.^30^ Different disease states may have specific miRNA states; thus, miRNAs in these specific states have the potential to be exploited as markers for disease diagnosis.

Although the study was successful, there are still some shortcomings, such as a small amount of sequencing samples and insufficient qPCR sample size. We hope that related studies with a larger sample size can corroborate our experimental results.

## Conclusion

miR-206, mi-R-126, and miR-222 significantly altered in the plasma of HCC patients, and they can screen patients at risk of HCC.

## Figures and Tables

**Table 1.** Clinical Features of the Study Population

Clinicopathological Feature	Number of Participants, n (%)	Groups	
HCC, n (%)	Control, n (%)
Age (years)			
Median (range)	59 (40-80)	60 (40-80)	56 (41-69)
Gender			
Male	39 (67.2)	26 (68.4)	13 (65)
Female	19 (32.8)	12 (31.6)	7 (35)
AFP			
>8.78	25 (43.1)	25 (65.8)	0 (0)
<8.78	33 (56.9)	13 (34.2)	20 (100)
AST: Aspartate aminotransferase			
>40	15 (25.9)	12 (31.6)	3 (15)
<40	43 (74.1)	26 (68.4)	17 (85)
ALT: Alanine aminotransferase			
>40	21 (36.2)	16 (42.1)	5 (25)
<40	37 (63.8)	22 (57.9)	15 (75)
Cirrhosis			
Positive	24 (41.4)	24 (63.2)	0 (0)
Negative	34 (58.6)	14 (36.8)	20 (100)
Hepatitis B			
Positive	25 (43.1)	25 (65.8)	0 (0)
Negative	33 (56.9)	13 (34.2)	20 (100)

**HCC, **hepatocellular carcinoma; **AFP, **α-fetoprotein.

**Table 2.** Discriminative Ability of Individual and Combined miRNAs (2^−ΔΔCt^ ) and AFP Between HCC and Control

Group	miRNA	Cut-off Value	Sensitivity (%)	Specificity (%)	AUC	SE	*P*
HCC versus control	miR-126	2.082	81.6	65	0.751	0.073	.001
	miR-222	2.207	55.3	90	0.686	0.071	.008
	miR-206	1.315	51.9	90	0.713	0.067	.002
	AFP	9.27	78.9	90	0.889	0.042	<.0001
	miR-126+miR-206	–	81.6	85	0.887	0.043	<.001
miR-126+miR-206+AFP	–	92.1	100	0.989	0.008	<.001

**HCC, **hepatocellular carcinoma; **AFP, **α-fetoprotein; AUC, area under the curve; SE, standard error; miRNA, microRNA.

**Table 3.** Simple and Multivariate Logistic Regression Analysis to Identify Independent Risk Factors for the Development of HCC

Parameters	Simple Logistic Regression		Multiple Logistic Regression	
OR 95% CI	*P*	OR 95% CI	*P*
miR-206	2.953 1.156-7.542	.024	10.554 1.489-74.817	.018
miR-126	0.576 0.381-0.869	.009	0.216 0.067-0.698	.01
miR-222	1.745 1.036-2.940	.036	1.822 0.937-3.542	.077
miR-206+miR-126	443.942 (16.755-11763.007)	.00	5027.258(26.575-951007.481)	.001

**HCC, **hepatocellular carcinoma; miRNA, microRNA; OR, odds ratio.
